# Downhill Sections Are Crucial for Performance in Trail Running Ultramarathons—A Pacing Strategy Analysis

**DOI:** 10.3390/jfmk7040103

**Published:** 2022-11-21

**Authors:** Matteo Genitrini, Julian Fritz, Georg Zimmermann, Hermann Schwameder

**Affiliations:** 1 Department of Sport and Exercise Science, University of Salzburg, 5400 Hallein-Rif, Austria; 2 Adidas AG, 91074 Herzogenaurach, Germany; 3 Team Biostatistics and Big Medical Data, IDA Lab, 5020 Salzburg, Austria; 4 Research Management & Technology Transfer, Paracelsus Medical University, 5020 Salzburg, Austria

**Keywords:** trail running, pacing strategy, ultramarathons, inclined running

## Abstract

Trail running is an increasingly popular discipline, especially over long-distance races (>42.195 km). Pacing strategy, i.e., how athletes modulate running speed for managing their energies during a race, appears to have a significant impact on overall performance. The aims of this study were to investigate whether performance level, terrain (i.e., uphill or downhill) and race stage affect pacing strategy and whether any interactions between these factors are evident. Race data from four race courses, with multiple editions (total races = 16), were retrieved from their respective events websites. A linear mixed effect model was applied to the full dataset, as well as to two subgroups of the top 10 male and female finishers, to assess potential differences in pacing strategy (i.e., investigated in terms of relative speed). Better finishers (i.e., athletes ranking in the best positions) tend to run downhill sections at higher relative speeds and uphill sections at lower relative speeds than slower counterparts (*p* < 0.001). In the later race stages, the relative speed decrease is larger in downhill sections than in uphill ones (*p* < 0.001) and in downhill sections, slower finishers perform systematically worse than faster ones, but the performance difference (i.e., between slower and faster finishers) becomes significantly larger in the later race stages (*p* < 0.001). Among elite athletes, no difference in pacing strategy between faster and slower finishers was found (*p* > 0.05). Both men (*p* < 0.001) and women (*p* < 0.001), in the later race stages, slow down more in downhill sections than in uphill ones. Moreover, elite women tend to slow down more than men (*p* < 0.001) in the later race stages, regardless of the terrain, in contrast to previous studies focusing on road ultramarathons. In conclusion, running downhill sections at higher relative speeds, most likely due to less accentuated fatigue effects, as well as minimizing performance decrease in the later race stages in downhill sections, appears to be a hallmark of the better finishers.

## 1. Introduction

Trail running is a strongly emergent endurance running discipline [1]; it has been previously defined as any running taking place in open country on unpaved surfaces (i.e., off-road, with <20–25% paved surface [2]). Trail running competitions offer a number of possible distances, from 5 km up to several hundreds. The events that are by far the most attended are ultratrail races, which are trail runs longer than a traditional 42.195 km marathon. The 100 km and 100 miles races are particularly popular, which can host up to > 1500 runners per edition (http://www.utmbmontblanc.com/ (accessed on 1 December 2020)).

Prolonged running on steep terrains results in a heavy physical load, which has been the object of several investigations in both biomechanics and physiology.

Inclined running, in fact, results in altered spatiotemporal parameters, as well as in kinematics and kinetics, compared to level running [3]. For instance, uphill running is characterized mainly by a concentric phase, which results in higher power generation at the hip joint [4], and higher net mechanical work to increase the body’s potential energy [5]. Conversely, the eccentric phase is predominant in downhill running, resulting in higher power absorption at the knee and hip joints [6]. As a result, the concentric phase is approximately 3–5 times more energetically demanding than the eccentric one [7]. Therefore, the physiological cost of running (i.e., the amount of energy spent to transport the subject’s body over a given distance [8]) can be represented by a reversed J-shape as a function of slope [9]. In particular, it is proportional to inclines more positive than −10% and more negative than −20%. Between −10% and −20% the cost of running is minimized [9,10], with −16% being the slope where energy generation is minimized [11]. The different gradient profiles of ultratrail races therefore pose a significant energetic challenge to competitors.

An athlete’s pacing strategy (i.e., how an athlete distributes energy and work throughout an exercise task) has a significant impact on their ultratrail performance [12].

Positive pacing (i.e., slowing down as the race proceeds) has been reported in road ultramarathons and ultratrail races. This pace strategy is described as a so-called reverse J-shape, with an athlete slowing throughout the race except for during the final stage, the end spurt [13,14,15,16,17,18,19,20]. Alternatively, even pacing is characteristic of minimal speed variation across the whole race. It has been associated with better finish times in ultratrail races [14,21], and road ultramarathons [13,17].

Athletes taking part in trail running ultramarathons have different characteristics (e.g., performance level, sexes and age groups). Therefore, the influence of these factors on pacing strategy has been the object of previous pacing strategy/efficiency investigations. When considering performance level, previous studies found no influence on pacing strategy. It has been suggested that inter-athlete differences in fitness level were minor, compared to the high fitness level required to complete an ultratrail race [21].

When considering sex, women seem to exhibit more even pacing in road marathons and ultramarathons [18,22,23,24], whilst men employ a more even pace in ultratrail races [21]. However, several investigations also reported no difference between sexes [13,20].

When considering runner age, some investigations found no influence on pacing strategy in road ultramarathons [13,18], whilst others found that the fastest finishers in both road ultramarathons and ultratrail races are between 35 and 55 years old [25,26,27,28].

In addition to the athletes’ characteristics, the influence of extrinsic factors (e.g., terrain) was investigated with respect to pacing strategy in ultratrail races. On level terrains, speeds decrease mainly during the first half of the race, whilst for uphill and downhill sections, speeds decrease across the whole race [16].

This far, the relatively small number of studies published with respect to ultratrail running have focused on factors (e.g., gender, performance level, terrain and race stage) reported from either a single race or multiple editions of the same race [14,16,21,25,26,28]. Nonetheless, it remains unclear whether a specific pacing strategy is present in ultratrail races in general, rather than in *a certain* ultratrail race, especially when comparing faster finishers to slower finishers. A comprehensive analysis focusing on multiple races, thus leveraging a large amount of data from multiple race courses, as well as focusing on how multiple factors interact with each other influencing pacing strategy, would help shed light in this regard. Practical benefits would include not only valuable information for coaches and practitioners, but also support race organizers, e.g., the location of first aid points, understanding where less proficient athletes experience the most difficulty.

Therefore, the aim of this study was to quantify the influence of performance level, terrain and race stage, as well as their interaction with pacing strategy, by means of a multilevel statistical approach and considering multiple race courses and editions. 

## 2. Materials and Methods

### 2.1. Race and Athletes’ Data

Four popular races with online race data reported within the past 5 years were considered in the current analysis. Five editions of the Ultra Trail Mont Blanc (UTMB, 2015–2019), five editions of the Courmayeur-Champex-Chamonix (CCC, 2015–2019), three editions of the Javelina 100 km (2017–2019) and three editions of the Javelina 100 mi (2017–2019) were included. Only the three most recent editions were included from the Javelina 100 km and the Javelina 100 mi because data from previous editions were incomplete. In total, 4 courses and 16 races were included for subsequent analysis. Race data are publicly available at the following links.

CCC and UTMB: https://utmbmontblanc.com/cn/page/349/349.html (accessed on 1 December 2020); Javelina 100 km and 100 mi: https://aravaiparunning.com/network/javelinajundred/results/ (accessed on 1 December 2020).

Runner-specific data were retrieved, which included the athlete’s final ranking (position), section split times (seconds) and demographic information (sex). Athletes with incomplete datasets were not retained for further analysis. In total, 16,518 athletes (14,330 male, 2188 female) were included. Likewise, race course characteristics of the event, for every course and edition, were retrieved through their event guide from their respective official websites. The guide provided the checkpoint locations (expressed in km from the start), checkpoint altitude (in m) and the elevation profile of the race.

### 2.2. Data Analysis

The terrain was categorized as uphill, downhill or level based on the section between two consecutive checkpoints, according to the course elevation profile. Sections consisting of non-monotonic slopes (i.e., multiple different terrains between two consecutive checkpoints) were discarded, as well as those categorized as flat sections, because they were too sparse. Further, we established whether a section belonged to the first or second half of the race; when a section was completed entirely in the first half of the race (by distance), it was assigned to the first half of the race and in any other case it was assigned to the second half of the race. 

The average race speed (ARS) of every athlete was calculated by dividing the race distance by the finish time. The average speed of a section (ASS) for each section was calculated by dividing the distance between two consecutive checkpoints by the split time of the section. Pacing strategy was then quantified and investigated in terms of relative speed, i.e., the relative section speed percentage (RSS; the average section speed normalized to the average race speed) during incline or decline sections was obtained by dividing the ASS by the ARS, such that RSS = (ASS/ARS) × 100. Importantly, sections were excluded if they were characterized as mixed or flat. Therefore, the mean value of all retained RSS values for a given participant does not necessarily equal 100%. 

An athlete’s performance level was categorized as either the top or bottom 50% of finishers based on their rankings. The top ten athletes, hereafter referred to as elite athletes, were the ten best males and females of each race. They were subsequently divided in to two performance level groups, such that athletes who ranked from one to five were assigned to the top 50% and athletes that ranked from six to ten were assigned to the bottom 50%. Therefore, three datasets were modelled: full dataset (including all finishers), elite men (top 10 men from every race) and elite women (top 10 women from each course). 

### 2.3. Statistics

Linear mixed effect models (LMEMs) were performed in R (R Development Core Team, 2015) using the “lmertest” package [29] as previously described in works characterized by data with comparable multilevel structures [30,31]. LMEMs were chosen because the dataset containing all the participants was not balanced across the conditions (i.e., some race courses presented more finishers than others) and LMEMs are not sensitive to the effects of unbalanced datasets. Furthermore, various levels of dependencies in the data had to be taken into account (i.e., data were obtained from four different races with three or five editions). The resulting model was the best-fitting one for both the full dataset (i.e., all finishers) and the two elite datasets (i.e., elite males and elite females only). The dependent variable was RSS. The fixed effects were performance level (top or bottom 50%), terrain (uphill or downhill) and race half (first or second half of the race). Additionally, the three-way interaction between these fixed effects was included in the model. Fixed effects were set as categorical and contrast-coded; reference levels were the top 50% of finishers for ‘performance level’ (for the full and two elite datasets), downhill sections for ‘terrain’ and first half of the race for ‘race half’. Random intercepts for the different race courses (in which different editions were nested) and for the section length were included as random effects. Section length was also included as a random effect and categorized as long or short; the threshold was identified as the median value in the distribution of all section lengths present in the dataset. A model comparison was used to assess whether the inclusion of sex as a random effect would provide a better fit of the full dataset, according to the Akaike Information Criterion (AIC). We found that the inclusion of sex did not significantly improve the model (α = 0.05). 

The best-fitting model (the same for the three datasets) is reported, where f.e. indicates a fixed effect, and r.e. indicates a random effect:

RSS = Performance level (f.e.) × Terrain (f.e.) × Race half (f.e.) + race course/edition (r.e.) + section length (r.e.)

This was the maximal random-effects structure that converged. The inclusion of a random effect for the single athletes—nested into the race editions—did not allow model convergence, as the variance explained by this factor was almost null. For the final (best-fitting) model, the lmertest package was used to assess significance of each fixed effect and their interaction by means of a three factor ANOVA. Satterthwaite’s method [29] was used to calculate the denominator’s degrees of freedom, and alpha was set to 0.05. The 95% confidence intervals of the fixed effects are subsequently reported. 

A positive model estimate (β) indicates the reference level data are smaller than those of the non-reference level, whereas a negative estimate indicates that the reference level data are larger than those of the non-reference level. The value of the estimate indicates the difference between the two groups. All three models (i.e., full dataset, elite men and elite women) were validated by visually inspecting the normal distributions of residuals via both histograms and quantile-quantile plots (Q-Q plots).

## 3. Results

**Figure 1:**
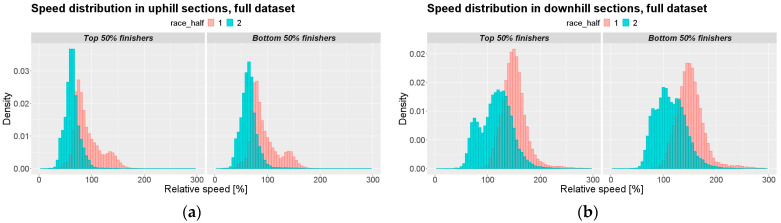

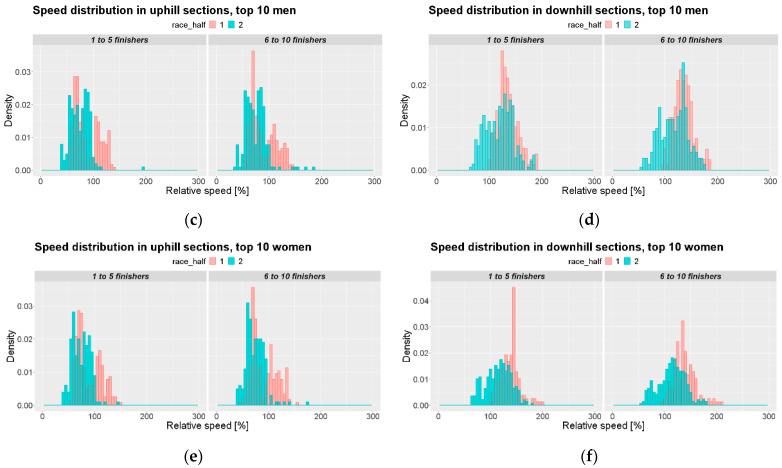
Relative speed section (RSS). Subfigures (**a**,**b**): full dataset; subfigures (**c**,**d**): top 10 men of all races; subfigures (**e**,**f**): top 10 women of all races. Left column: uphill sections; right column: downhill sections.

A total of 16,518 athletes (14,330 = male, 2188 = female) were included in the analysis. In total, 215,060 RSS observations were included. The RSS distributions are reported in Figure 1.

### 3.1. Full Dataset

Model coefficients for the full dataset are reported in Table 1.

**Figure 2:**
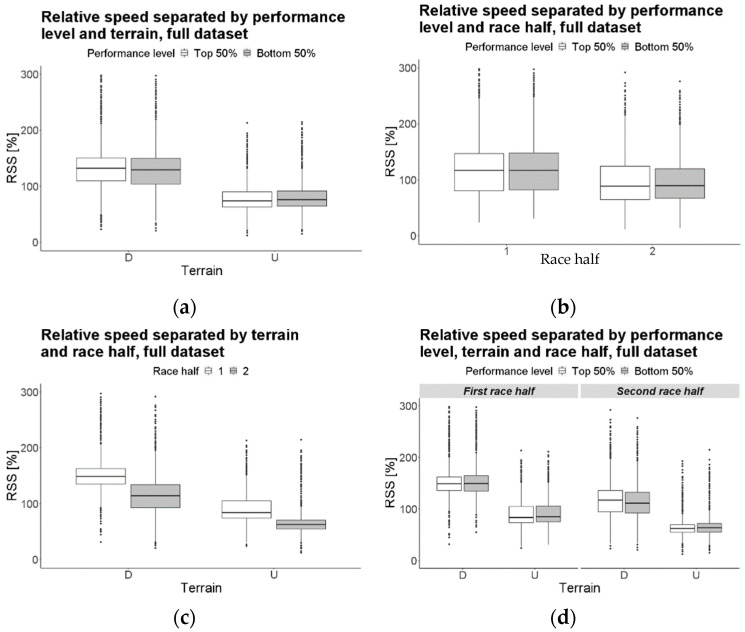
Boxplots representing the three two-way interactions (**a**–**c**) and the three-way interaction (**d**) of the full dataset model.

The RSS of the bottom 50% of finishers was 0.54% higher than that of the top 50% finishers, when considering data from all sections. The RSS for uphill sections was lower (−57.03%) than that for downhill sections. The RSS in the second race half was lower (−33.29%) than in the first half. The difference in the RSS between the different terrains was 2.62% larger for the top 50% of finishers than for the bottom 50% (Figure 2a). The RSS decrease in the second race half was lower (−1.71%) for the top 50% of finishers than for the bottom 50% of finishers (Figure 2b). The RSS decrease in the second race half was 24.46% larger for downhill sections than for the uphill ones (Figure 2c). Furthermore, the trend is 3.07% larger for the bottom 50% of finishers (Figure 2d).

### 3.2. Elite Men and Women

**Figure 3:**
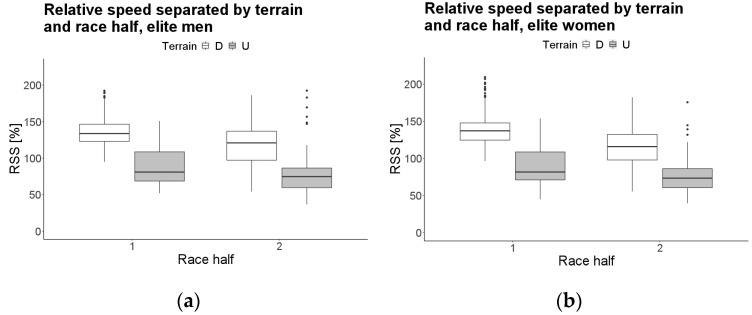
Boxplots representing the two-way interactions between terrain and race half for the elite dataset models, male (**a**) and female (**b**).

Model coefficients for elite men and women are reported in Table 2 and Table 3, respectively. The RSS for the uphill sections was lower than for the downhill ones (−48.43% for men, −47.18% for women). RSS in the second race half was slower than in the first race half (−17.87% for men, −22.37% for women). In the second race half, the RSS decrease was larger for the downhill sections than for the uphill ones (Figure 3; 15.87% for men, 20.04% for women).

## 4. Discussion

### 4.1. Full Dataset

Concerning the main effect on performance level, the RSS was significantly, albeit slightly, higher for the bottom 50% of finishers (0.54% higher than the top 50% of finishers), as seen in Table 1. Previous work has shown that slower finishers tend to reduce their speed more than their faster counterparts in the final race stages, when their speeds were normalized to their speeds at the beginning of the race [28]; this is in line with our findings, because the RSS (i.e., normalized to the ARS) implies that slower runners would present higher relative speeds in the early sections, which contributes to a slightly higher mean value across all sections for slower finishers. 

Not surprisingly, relative speeds for the uphill sections were remarkably lower than for the downhill ones (−57.03%), which is likely due to the higher physiological demand of uphill running [9]. When considering race stages, relative speed in the second half was lower than in the first half (−33.29%). This is consistent with previous findings for track disciplines, as well as marathons and ultratrail races [12,13,14,15,16,17,18,19,20,32]. It has been shown that self-selected exercise intensity decreases progressively in endurance events longer than four hours [33,34]. Abbis and colleagues (2008) explain that this trend may be the consequence of increased glycogen depletion [35,36] resulting in altered substrate utilization [37,38,39], neuromuscular fatigue [38,40,41] and psychological factors associated with fatigue. 

A significant interaction between performance level and terrain indicates that the difference in the RSS between downhill and uphill sections is larger for the top 50% of finishers compared to the bottom 50% of finishers (2.62%; Figure 2a); faster finishers tended to deliver a higher effort in the downhill sections, most likely due not only to their superior capacity to cope with the technical difficulty of such an incline, but also, most importantly, due to the less accentuated exercise-induced muscle damage and fatigue, which are effects resulting from systematic exposure to downhill running during training [42]. This results in a larger RSS difference between the different terrains. To the best of our knowledge, we are the first to report this finding in ultratrail races. These findings support previous work that considered short trail running races (<20 km), which found that descent skills (i.e., those qualities needed for fast descent but not for fast ascent) were significantly correlated to better finish times [43]. 

A significant interaction between performance level and race half indicates that the decrease in the RSS in the second race half was slightly smaller (−1.71%) for the top 50% of finishers (Figure 2b). Slower finishers slowed down more in the later race stages compared to their faster counterparts, when including data from both uphill and downhill terrains. These findings agree with those previously reported, in which, slower runners exhibited a larger decrease in performance during the later race stages compared to the early ones [28]. 

A significant interaction between terrain and race half indicates that the difference in the RSS between the downhill and uphill sections is larger (24.46%) in the first race half. In the second race half, the RSS difference between the downhill and uphill sections becomes smaller because athletes tend to slow down more in the downhill sections than in the uphill ones, when considering data of both performance levels (Figure 2c). In fact, both the downhill and uphill sections show lower values in the second race half; a smaller difference in the second race half indicates that, despite the RSS decreasing for both terrains, the decrease was larger in the downhill sections than in the uphill ones. This outcome is consistent with that of other authors [16], who, nonetheless, investigated pacing strategy of only 15 male athletes during a single ultratrail race; the current work generalizes such a tendency from a restricted group of athletes to the population (both sexes) of multiple race courses. 

As previously explained, a significant interaction between performance level and terrain indicates that the difference in the RSS between the downhill and uphill sections is larger for the top 50% of finishers compared to the bottom 50% of finishers (i.e., better finishers run faster downhill), most likely due to their more effective physical adaptations to such terrain, whereas it is plausible that slower runners focus mainly on the uphill sections, as positive inclines are metabolically more demanding [9]. A significant 3-way interaction means that the aforementioned difference in pacing strategy, between faster and slower finishers, becomes significantly larger (3.07%) in the second race half; the faster finishers appear to be better adapted to negative slopes, so as the race progresses, they deliver better performances in the downhill sections, compared to their slower counterparts (Figure 2d).

### 4.2. Top 10 Men and Women

The RSS in the uphill sections was lower than in the downhill ones for both sexes (−48.43% for men, −47.18% for women). The same was found with respect to race stages, where the relative speed in the second half was significantly lower than in the first half. The elite female runners had a larger RSS decrease compared to males, which is supported by a more accentuated positive pacing (−17.87%for men, −22.37% for women). In this sense, the less even pacing showed by women agrees with some previous findings regarding ultratrail races [21], which report higher pace variation for women. Nonetheless, previous studies focusing on road races have reported an opposite trend, with women pacing more evenly than men [13,18,22,24,32,44]. Among the possible reasons for this discrepancy, Suter and colleagues (2020) [21] hypothesized that men are potentially able to run uphill faster due to their higher muscle mass to fat mass ratio [45,46,47]. This characteristic would mean men are able to show a less accentuated decrease in performance in uphill traits in the later race stages, compared to their female counterparts, i.e., a “less positive” pacing. This hypothesis is supported by previous research findings correlating trail running performance to VO2max and fat mass in both male and female athletes [48,49,50].

A significant interaction between terrain and race half was found, indicating that the difference in the RSS between the downhill and uphill sections is larger in the first race half; in the second race half the RSS difference between the downhill and uphill sections becomes smaller because athletes tend to decrease their RSS more in the downhill sections than in the uphill ones (Figure 3a,b). This corroborates previous findings [16], where a greater speed decrease was observed in the downhill and flat sections, compared to uphill; the present study extends them to the subgroups of elite athletes and both sexes. Furthermore, such a trend appears more clearly for women, who decreased their RSS by ~5% more than men (15.87% for men, 20.04% for women) from the first to the second half of the race. Suter and colleagues (2020) [21] hypothesized that a possible explanation for why women perform worse in later race stages may be that their body composition is different with respect to men, which results in a more accentuated speed decrease in the uphill sections. To add to these observations, we hypothesize that the main cause for worse performance by female elites in later race stages may be because of the downhill sections; when experiencing fatigue, the more technical downhill sections might have a much higher fall risk than the uphill ones, given the higher running speeds. In this sense, sex differences in confidence level, attitude towards risk and competitiveness [18,23,32] may induce males to deliver a higher effort in the downhill sections despite the risk and fatigue, compared to females. These psychology-related observations should be summed to the ones above (Suter 2020, [21]), as for physiological determinants, so explaining both the more accentuated interaction between terrain and race half and the main effect of race half for female elites. No significant differences were found regarding performance level as a main effect or as an interaction, meaning that no difference in pacing strategy between the top five runners and those athletes placing six to ten was detected. This outcome suggests that the performance difference among top performers is determined by other factors than the pacing strategy (e.g., VO2max fat mass, body mass index [50] or lactate threshold [49]). As for downhill running, the capacity to minimize exercise-induced muscle damage during competitions by means of specific training plays a critical role [42], as well as the musculotendinous capacity to absorb impacts as high as 6G [51].

## 5. Conclusions

To the best of our knowledge, the present study is the first one focusing exclusively on long-distance trail running competitions (including multiple race courses and editions) while analyzing the effects of performance level, terrain and race stage on pacing strategy. Moreover, our study utilized a large (>15 k) sample of athletes. The novel findings of the present study are: (i) faster finishers tended to run the downhill sections faster and the uphill sections slower, compared to their slower counterparts, (ii) the trend described in (i) increases significantly in later race stages, with differences in pacing strategy between faster and slower finishers becoming more accentuated, (iii) there was a general tendency to slow down more in the downhill sections than in the uphill ones in the later race stages, (iv) among elite athletes, no differences in pacing strategy were present. Specific to elite athletes, both male and female athletes tended to slow down more in the downhill sections compared to the uphill ones in the later race stages. In summary, the capacity to deliver a higher effort in the downhill sections, as well as the capacity to limit performance decrease in the downhill sections in the later race stages, appears as a determinant of better trail race finish placements. For elite athletes, no difference in pacing strategy was observed between faster and slower finishers, with women pacing less evenly than men and slowing down more in the downhill sections in the later race stages. Future studies should focus on how terrain steepness affects pacing strategy, i.e., steeper or milder uphill/downhill traits, as well as on the role played by flat sections on overall performance in ultratrail races. 

## 6. Limitations

As previously mentioned, it was not possible to retain flat sections, as those were present only in very few instances of our 16 included ultratrail races. On the other hand, just because this terrain was so scarcely present, the accuracy of our model was not compromised.

Similarly, it was not possible to include the steepness of the uphill and downhill sections (e.g., 5%, 10% etc.) in the model, as such data were not uniformly present across race courses and race stages, which would have weakened any conclusion as far as steepness is concerned and resulted in a lack of generalizability.

## Figures and Tables

**Table 1:** Linear mixed effect model output for the full dataset; significant results are highlighted in bold.

*Predictors*	*Estimates*	*SE*	*CI*	*p*
performance level	0.54	0.10	0.35–0.72	**<0.001**
terrain	−57.03	0.10	−57.22–−56.84	**<0.001**
race half	−33.29	0.10	−33.48–−33.10	**<0.001**
performance level * terrain	2.62	0.19	2.25–2.99	**<0.001**
performance level * race half	−1.71	0.19	−2.08–−1.34	**<0.001**
terrain * race half	24.46	0.20	24.06–24.85	**<0.001**
performance level * terrain * race half	3.07	0.38	2.32–3.81	**<0.001**

**Table 2:** Linear mixed effect model output for the top 10 men; significant results are highlighted in bold.

*Predictors*	*Estimates*	*SE*	*CI*	*p*
performance level	−0.26	0.99	−2.19–1.67	0.793
terrain	−48.43	1.01	−50.40–−46.45	**<0.001**
race half	−17.87	1.02	−19.87–−15.88	**<0.001**
performance level * terrain	1.82	1.97	−2.04–5.68	0.355
performance level * race half	−1.46	1.97	−5.32–2.40	0.459
terrain * race half	15.87	2.06	11.83–19.91	**<0.001**
performance level * terrain * race half	1.55	3.94	−6.17–9.26	0.695

**Table 3:** Linear mixed effect model output for the top 10 women; significant results are highlighted in bold.

*Predictors*	*Estimates*	*SE*	*CI*	*p*
performance level	−0.18	1.02	−2.18–1.83	0.864
terrain	−47.18	1.05	−49.23–−45.13	**<0.001**
race half	−22.37	1.06	−24.45–−20.29	**<0.001**
performance level * terrain	0.33	2.05	−3.69–4.34	0.874
performance level * race half	−2.45	2.05	−6.47–1.56	0.231
terrain * race half	20.04	2.14	15.84–24.24	**<0.001**
performance level * terrain * race half	−1.43	4.10	−9.46–6.60	0.727

## Data Availability

Data are available upon request to the authors.
